# Conservation physiology of animal migration

**DOI:** 10.1093/conphys/cov072

**Published:** 2016-02-29

**Authors:** Robert J. Lennox, Jacqueline M. Chapman, Christopher M. Souliere, Christian Tudorache, Martin Wikelski, Julian D. Metcalfe, Steven J. Cooke

**Affiliations:** 1 Fish Ecology and Conservation Physiology Laboratory, Department of Biology, Carleton University, 1125 Colonel By Drive, Ottawa, ON, Canada K1S 5B6; 2 Department of Biology, Carleton University, 1125 Colonel By Drive, Ottawa, ON, Canada K1S 5B6; 3 The Sylvius Laboratory, Institute of Biology, Leiden University, Sylviusweg 72, Leiden 2333 BE, The Netherlands; 4 Department of Migration and Immuno-ecology, Max-Planck Institute for Ornithology, Radolfzell, Germany; 5 Department of Biology, University of Konstanz, Konstanz, Germany; 6 Centre for Environment, Fisheries and Aquaculture Science (Cefas), Lowestoft Laboratory, Suffolk NR33 0HT, UK; 7 Institute of Environmental Science, Carleton University, Ottawa, ON, Canada K1S 5B6

**Keywords:** Behaviour, energetics, human impacts, mechanism, movement

## Abstract

Migration is a widespread phenomenon among many taxa. This complex behaviour enables animals to exploit many temporally productive and spatially discrete habitats to accrue various fitness benefits (e.g. growth, reproduction, predator avoidance). Human activities and global environmental change represent potential threats to migrating animals (from individuals to species), and research is underway to understand mechanisms that control migration and how migration responds to modern challenges. Focusing on behavioural and physiological aspects of migration can help to provide better understanding, management and conservation of migratory populations. Here, we highlight different physiological, behavioural and biomechanical aspects of animal migration that will help us to understand how migratory animals interact with current and future anthropogenic threats. We are in the early stages of a changing planet, and our understanding of how physiology is linked to the persistence of migratory animals is still developing; therefore, we regard the following questions as being central to the conservation physiology of animal migrations. Will climate change influence the energetic costs of migration? Will shifting temperatures change the annual clocks of migrating animals? Will anthropogenic influences have an effect on orientation during migration? Will increased anthropogenic alteration of migration stopover sites/migration corridors affect the stress physiology of migrating animals? Can physiological knowledge be used to identify strategies for facilitating the movement of animals? Our synthesis reveals that given the inherent challenges of migration, additional stressors derived from altered environments (e.g. climate change, physical habitat alteration, light pollution) or interaction with human infrastructure (e.g. wind or hydrokinetic turbines, dams) or activities (e.g. fisheries) could lead to long-term changes to migratory phenotypes. However, uncertainty remains because of the complexity of biological systems, the inherently dynamic nature of the environment and the scale at which many migrations occur and associated threats operate, necessitating improved integration of physiological approaches to the conservation of migratory animals.

## Abstract (precis)

Conservation physiology has great potential to help us understand how migratory animals interact with current and future anthropogenic threats. Migration is inherently challenging such that additional stressors derived from altered environments or interaction with human infrastructure or activities could lead to long-term changes to migratory phenotypes.

## Introduction

Migration is one of nature's most captivating phenomena. Migratory movements can be as vast as the transcontinental treks of African wildebeest or as minute as diel vertical migration of zooplankton within metres of the water surface. Movement is an inextricable component of animal behaviour, but migration is distinct from dispersal or station-keeping behaviours because it is predictable, directional and persistent (Dingle and Drake, 2007), owing to physiological changes that underlie the migratory life stage. Migration is exhibited by every major animal taxon and, ultimately, maximizes survival and reproductive success through the utilization of key habitats, food sources and breeding grounds and/or the avoidance of adverse environmental conditions (Dingle and Drake, 2007). The movement of large numbers of animals from one region to another can benefit ecosystems by linking nutrient sources and increasing local diversity; these factors may increase ecosystem resilience in times of disturbance (Bauer and Hoye, 2014). Conversely, the unique physiological demands of migration may leave migratory species more susceptible to disturbance (Wilcove and Wikelski, 2008).

Life-history phenotypes such as migration are expressed through behaviours exhibited by individuals in response to internal changes to their physiology (Ricklefs and Wikelski, 2002). Physiology therefore plays an important and foundational role in animal migration, and migratory physiology is a mechanistic sub-discipline of migration and movement biology (Bowlin *et al*., 2010; Dingle, 2014; Jachowski and Singh, 2015). Changes to the central nervous system characterize migration, because migrating animals are undistracted by cues that would otherwise elicit vegetative responses (Dingle, 1996). Moreover, physiological mechanisms control the timing, locomotion and synchronicity of migration, which dictate migratory behaviour and ultimate success (Wingfield *et al*., 1990). Migration is composed of complex and dynamic interactions among individual genetics, behaviour, physiology, biomechanics and the environment (Dingle, 2006). In addition, migrations are inherently challenging; large-scale movement across complex landscapes requires vast amounts of energy (Wikelski *et al*., 2003; Bowlin *et al*., 2005; Bishop *et al*., 2015). Furthermore, some species conduct migrations without interruption for refuelling, working off fixed energy reserves (Stephens *et al*., 2009). Given that a failed migration directly affects lifetime fitness of individuals (Dingle, 1980), natural selection has the potential to alter populations and migratory phenotypes rapidly. In some cases, this can lead to changes in population structure, evolutionary bottlenecks, inbreeding depression and extirpation or extinction (Wilcove and Wikelski, 2008), which have broader impacts on animal communities and entire ecosystems.

Over the last several decades, global changes and biodiversity losses have created a challenging landscape for conservation science. Climate change, habitat alteration, species invasions and pollution are altering landscapes and creating new challenges for animals. Migratory species represent a unique challenge because of their high mobility and their reliance on multiple habitats to complete their life history, meaning that they may be subject to multiple and varied threats in different habitats such that predicting and understanding their ability to adapt is difficult (Robinson *et al*., 2009; Sih *et al.*, 2011; Gienapp, 2010). Conservation is a varied and dynamic science, the goals of which extend beyond simply avoiding extinction risk to understanding and conserving the traits and attributes of species that make them successful (Redford *et al*., 2011). Novel methodologies and solutions are constantly developing in an effort to achieve conservation objectives, including an increasing synergy between conservation and physiology (Wikelski and Cooke, 2006; Coristine *et al*., 2014; Lennox and Cooke, 2014). Conservation physiology focuses on understanding and predicting the responses of animals to environmental change and the potential for solving diverse conservation problems using physiological knowledge, approaches and tools (Cooke *et al*., 2013a,b). Given the importance of physiological mechanisms to animal migration, there are opportunities to implement physiology to enhance our understanding of migratory species and populations as well as develop novel conservation approaches that are informed by animal physiology. Here, we review the physiology of animal migration and demonstrate conservation physiology approaches for future research on human-induced environmental changes focused on key conservation questions where conservation physiology has the potential to play an important role. Although we consider all animal taxa in our review, the conservation physiology of migration literature is disproportionately rich in studies focused on fish and birds, which is reflected to some extent in the coverage below.

## Review

### Orientation and navigation

**Figure 1:**
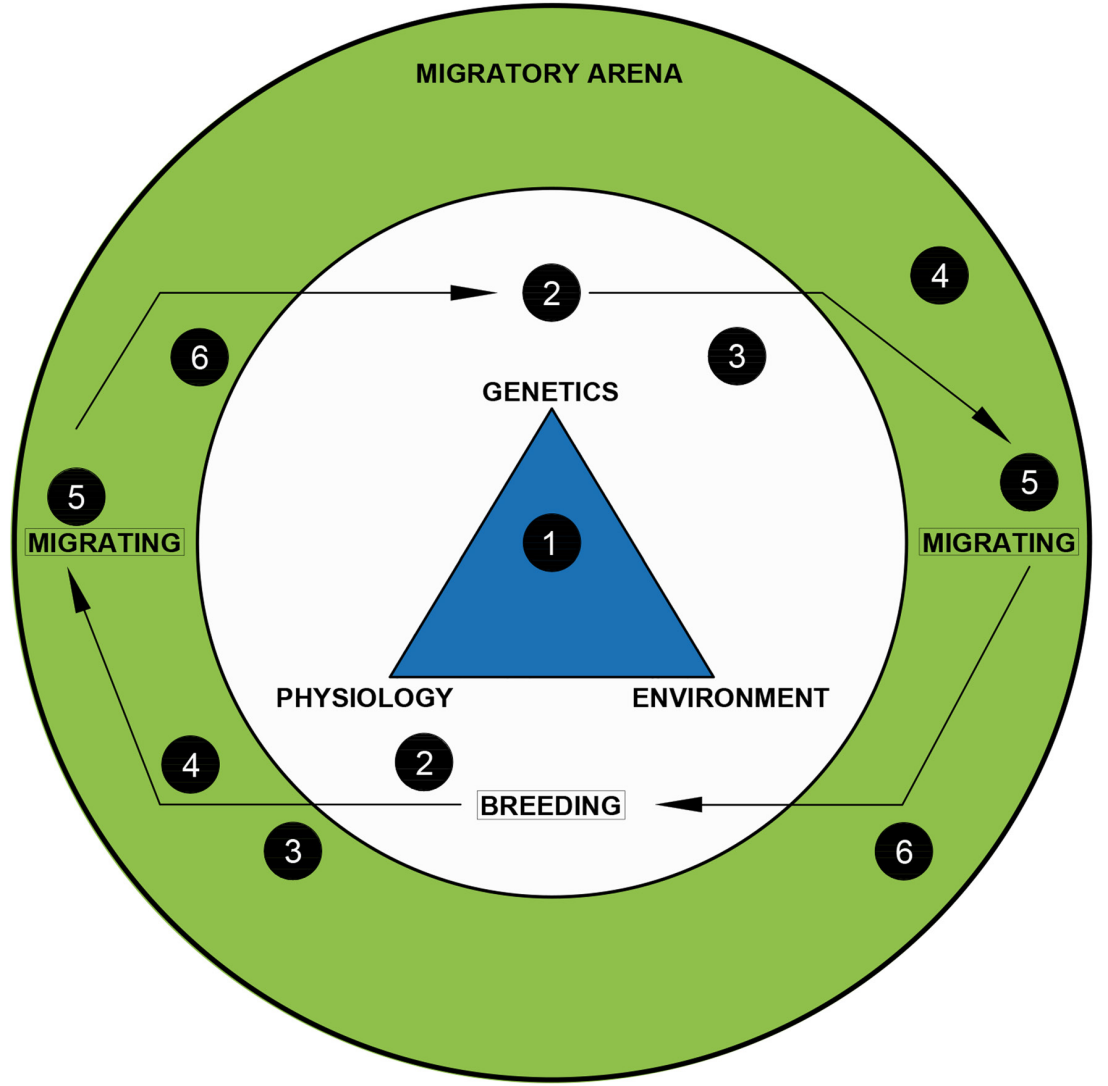
Migration is a suite of physiological changes that manifest as predictable, persistent, oriented movement of animals between environments in order to exploit seasonal productivity and maximize fitness. Genetics, physiology (including metabolism and condition) and environmental conditions can influence transition to the migratory life stage (1). Prior to entering the migratory arena, animals accumulate fuel, and their bodies often undergo physiological remodelling to reduce the cost of transport during migration, including atrophy of some organs and hypertrophy of exercise muscles or organs (2). The timing of migration is synchronized with distant environmental conditions via hormonal regulation, with an important role being played by melatonin produced by the photosensitive pineal gland in response to changing day lengths (3). Upon departure, animals use a variety of behavioural strategies to maximize the energetic efficiency, including soaring or gliding (4), as well as periodic stopovers to replenish energy stores (5). To find their target habitat, migrating animals have a variety of strategies for orienting, wayfinding and interpreting visual, olfactory and other sensory information from the environment that indicates their proximity to high-quality habitat (6). Once migrating animals reach their target habitat, they exit the migratory arena and resume vegetative behaviour.

The success of migration depends on an animal's ability to orient and navigate along migratory paths and requires physiological mechanisms for taking the best migratory route (Åkesson and Hedenström, 2007; Bauer *et al*., 2011; Fig. 1). Birds (Mouritsen and Hore, 2012), sea turtles (Lohmann 1991; Luschi *et al*., 2007), bats (Holland *et al*., 2006; Tian *et al*., 2010), salamanders (Phillips and Borland, 1992) and salmon (Putman *et al*., 2014) are among species that use magnetic signals for orientation (Wiltschko and Wiltschko, 2005; Lohmann *et al*., 2007, 2008). Cellular mechanisms supporting magnetoreception have not been unequivocally demonstrated to date (Tian *et al*., 2010; Edelman *et al*., 2015), but magnetite integrated into sensory tissue has been located in bird beaks (Fleissner *et al*., 2003), salamander thymoids and turtle heads. Magnetic orientation is usually used to orient at long distances, such as in the open ocean (Putman *et al.*, 2013, 2014). Long-distance navigation can be disturbed by a variety of human developments that create or modify magnetic signatures used by animals. The specific factors that affect animal navigation or homing depend on the navigation techniques used by the animal. There is some evidence that geomagnetic detection by birds can be disrupted by magnetic fields created around cities (Ritz *et al*., 2004, 2009; Engels *et al*., 2014). However, evidence that this is occurring in the wild is lacking.

Light from the sun and other celestial bodies can be entrained by migrating animals for orientation (Able, 1982). Specifically, animals can detect polarized light from the sun to correct their movement path (Helbig, 1991; Reppert *et al*., 2004). Artificial lights can distract animals from their movement path (e.g. Dacke *et al*., 2003; Mazor *et al*., 2013); for example, beach, street and pathway lighting can entrain hatchling sea turtles, resulting in fewer successful migrations to the sea and reduced recruitment (Tuxbury and Salmon, 2005). Furthermore, seabirds can be disoriented at night when exposed to artificial light sources such as ships, lighthouses and oil and gas platforms, often ending in collision between the bird and the structure (Montevecchi, 2006). Light pollution from buildings, ships, aeroplanes and other structures has the potential to distract and disorient migrating animals and can increase the risk of death via collision or exhaustion when animals follow lights indefinitely (Jones and Francis, 2003; Merkel, 2010).

At close range, migrating animals generally rely on olfactory or visual cues to locate fine-scale areas in a habitat by searching for landmarks or appraising habitat qualities (Lohmann *et al*., 2008; Ueda, 2012). Experiments disrupting visual and olfactory pathways in migrating *Oncorhynchus masou* in coastal regions demonstrated a reduced ability to locate and enter natal streams (Ueda *et al*., 2000). Likewise, sea turtles use water- and airborne olfactory cues, which are believed to provide a source of navigational information throughout migrations (Koch *et al*., 1969; Lohmann *et al*., 2008). Turtle species that demonstrate high nesting site fidelity are thought to imprint on chemical gradients from natal grounds to guide reproductive migrations (Hasler and Scholz, 1983; Endres and Lohmann, 2013). Olfactory systems can also detect conspecific pheromones; indeed, red-sided garter snakes (*Thamnophis radix parietalis*) use these chemical signatures to follow movements of conspecifics to feeding areas and hibernacula and to locate partners during vernal breeding migrations (Lemaster *et al*., 2001). Alteration of chemical signatures in target habitats can mask or dilute chemical cues, causing animals to lose track of scents and become lost on migration. Acidification of inland waters from acid rain or pollution completely stopped further upstream migration of sockeye salmon (*Oncorhynchus nerka*; Ikuta *et al*., 2001). Stormwater runoff from roadways flushes chemicals into rivers, some of which (e.g. copper from brake pads) can impair olfactory sensitivity in coho salmon (*Oncorhynchus kisutch*) after even temporary exposure (McIntyre *et al*., 2008), perhaps interfering with navigation. Changes to water flow from redirection of water associated with irrigation, hydroelectric power generation and/or infilling of headwaters for development can redistribute or dilute chemical signatures, reducing the ability of aquatic animals to navigate, make successful migrations and recruit (Sato *et al*., 2000; Burnett *et al*., 2014a).

### Energetics

Migratory species travelling long distances between habitats require adaptations to optimize energetic output. Endurance during migration is a function of energy availability; therefore, accumulation of fuel is an essential mechanism supporting migration (Fig. 1). Fatty acids are the most efficient fuel source per unit weight and are important for reducing the cost of transport for migrating animals (Butler and Woakes 1990; Jenni and Jenni-Eiermann, 1998; McWilliams *et al*., 2004; Del Raye *et al*., 2013). To accumulate fuel, animals can pre-empt migration with hyperphagia, dietary changes and/or increased food assimilation efficiency (Bairlein, 2002; Santra *et al*., 2008). However, animals preparing to migrate must have access to food of sufficient quality and quantity in order to execute migration; therefore, identification and conservation of key food sources and habitat types is essential (e.g. Alonso-Mejía *et al*., 1997). When key habitats and food sources are degraded or lost, actions such as supplemental feeding or enrichment may mitigate impacts on migratory populations. To reduce maintenance costs during the migration, atrophy of non-essential organs (e.g. alimentary; Piersma and Lindström, 1997; Piersma *et al*., 1999) and hypertrophy of locomotory and cardiac muscle (Piersma *et al*., 1999; King *et al*., 2015) take place. To compensate for low metabolism, reptiles may circulate thyroxine (Southwood and Avens, 2010) to increase oxygen consumption, heart mass and metabolic enzyme activity, suggesting an endocrine role in facilitating migratory activity (see also Bishop *et al*., 1995). Summer growing seasons are extending and winters are shortening, which can prolong residence at summering grounds, increase risk of pathogen incubation (e.g. Bartel *et al*., 2011) and reduce the time available for the essential preparatory process of feeding and establishing fuel stores, resulting in decreased energy available for migration (e.g. beluga whale, *Delphinapterus leucas*; Bailleul *et al*., 2012). Ensuring protection of or supplementing existing habitats necessary for animals preparing to migrate might be beneficial for conserving migrants; for example, Masero (2003) found that anthropogenic salinas in Spain provided valuable replacement habitat for shorebirds initiating hyperphagia prior to migration. Increased temperatures result in higher costs of activity during migration; activity in warm temperatures increases cardiac stress and limits distribution of oxygen to tissues (e.g. Pörtner and Farrell, 2008; Eliason *et al*., 2013). Correspondingly, there is a need to understand better how plasticity and evolution in animals under thermal stress contribute to resilience (Anttila *et al*., 2014). Rapid dehydration can occur when birds experience warm temperatures, increasing the need for stopover during migration and delaying arrival (McKechnie and Wolf, 2009), potentially necessitating the protection of larger tracts of land that are important for stopover. In fact, some species may become incapable of or lose their will to move when temperatures are high, which can delay migration and result in mismatched timing of arrival relative to peak environmental conditions (e.g. fish: Baisez *et al*., 2011; Eliason *et al*., 2013; mammals: Post and Forchhammer, 2008).

Behavioural strategies are combined with physiological mechanisms to limit the cost of transport and maximize distance that it is possible to travel per unit of fuel. During migration, animals exploit wind and ocean currents for conveyance along the migration path (Wikelski *et al*., 2006). In addition, animals may maintain activity near to but not exceeding their upper aerobic limit to sustain endurance; such findings can contribute to improved design and management of fish passage structures at dams for fish migrating to and from reproductive sites (Burnett *et al*., 2014b; Silva *et al*., 2015). Given that it is costly to transport large amounts of fuel along migration, some species interrupt migration to refuel (Alerstam *et al*., 2003; Dingle and Drake, 2007; Sawyer and Kauffman, 2011). The need to feed during migration makes movement paths somewhat predictable and can allow for protected areas or strategic shipping/aircraft routes. Indeed, patterns of foraging behaviour and corresponding dive physiology of sea turtles moving to and from nesting habitat can allow for better management of shipping operations (e.g. Eckert *et al*., 1989; Plot *et al*., 2015). Other animals, such as birds and whales, make stopovers in highly productive feeding areas to refuel. Stopover time is influenced by food availability, fuel load and the rate of fuel deposition (Hedenström and Alerstam, 1997; Eikenaar and Bairlein, 2014). At stopover sites, birds will restore their alimentary organs, feed quickly and then re-atrophy the organs prior to departure. However, other animals spend much of their migration at stopover sites, allowing them to maximize energy intake and migrate synchronously with plant phenology (Mate *et al*., 2011; Jones *et al*., 2014; Sawyer *et al*., 2009). Urbanization and habitat degradation are affecting the availability of the key stopover sites where animals replenish energy stores, and the fragmented habitats can exacerbate stress (Ellis *et al*., 2012). A lack of suitable stopover habitat can exhaust the energy available for migration (Faaborg *et al*., 2010; Braithwaite *et al*., 2015) or might concentrate many individuals at some rare, productive islands in a landscape. Stopover habitats are disappearing, and establishment of alternative stopover sites (Garaita and Arizaga 2015) or the use of supplemental feeding (Jones *et al*., 2014) may be necessary to conserve migratory animals. Although natural areas offer many benefits to animals, Liu and Swanson (2014) found that birds using modified habitat did not have higher stress than those using natural habitat, indicating the potential for adaptation to the loss of natural stopover habitat. Nonetheless, continued reduction and replacement of natural stopover habitat for migrants could encourage mass aggregations, which can rapidly deplete the available resources and increase disease transmission among individuals (Altizer *et al*., 2011). For this reason, immunology is an increasingly important aspect of the conservation physiology of migratory animals (e.g. Mallory *et al*., 2015). Ultimately, understanding the importance of stopover habitats and their role in replenishing fuel provides necessary information for conservation. Bonter *et al*. (2007) inferred the importance, and thereby the conservation priority, of migratory bird corridor habitats by measuring the body condition of birds and identifying the most important sites. Whitlock *et al*. (2015) further suggested seasonal protection of key foraging habitats for Pacific bluefin tuna (*Thunnus orientalis*) based on observations of high energy intake in certain hotspots of the Pacific Ocean.

### Timing

The timing of migration exerts a considerable influence on fitness because it affects resource availability. Properly timed migration is important for avoiding unfavourable conditions and arriving at stopover sites and the ultimate destination at an appropriate time when environmental conditions are suitable. Mismatched timing may result in migrations coinciding with depleted food sources at stopover sites or reduced breeding opportunities at the destination (Meltofte, 1985). The timing of migration is somewhat determined by genetics (Berthold, 1996) and circadian/circannual biorhythms; however, the environment exerts a secondary influence on migration (Richardson, 1990; Fig. 1). Together, biotic and abiotic cues combine to control the endocrine system of migratory animals, which regulates the physiological and morphological changes necessary to prepare for departure, locomotion and arrival (Fig. 1).

Preparation for migration begins prior to departure and ensures that energetic reserves are sufficient for the journey (Ramenofsky and Wingfield, 2007). Consequently, departure is influenced in part by fuel reserves and body condition (Brodersen *et al*., 2008), which cue the release of behaviour-mediating hormones. In insects, juvenile hormone is the principal endocrine cue for initiating migration behaviour (Chapman *et al*., 2015). In vertebrates, fluxes in melatonin influence preparation for migration. The photosensitive pineal gland entrains information about photoperiod and controls melatonin secretion, which tracks circadian and circannual changes (Bradshaw and Holzapfel, 2007; Tosches *et al*., 2014; Winkler *et al*., 2014). In turn, melatonin stimulates androgen production (Crossin *et al*., 2010), a primary cue for the breeding migration of fish and birds (Wingfield *et al*., 1990). Among birds, melatonin concentrations regulate migratory restlessness (i.e. Zugunruhe) and a transition to nocturnal activity prior to departure (Gwinner, 1996). In addition to melatonin, fluxes of glucocorticoids, catecholamines, thyroxine, prolactin and leptin contribute to the timing of migration (Cornelius *et al*., 2013).

An important consequence of relying on fixed signals, such as photoperiod, is that changes to the climate result in the temporal mismatch of key life-history events (e.g. migration, breeding) of migratory animals from suitable environmental conditions (e.g. plant flowering, insect emergence). However, there is evidence of plasticity in the timing of migration because birds can adjust their migration timing, for example to compensate for poor weather (Richardson, 1990; Cochran and Wikelski 2005; Ramenofsky, 2011). Nonetheless, there are limits to such plasticity (DeWitt *et al*., 1998), and climate change may advance too rapidly for plasticity to compensate (e.g. Gauthier *et al*., 2013). Reed *et al.* (2011) predicted that natural advances in the timing of migration are likely to facilitate persistence of salmon, and efforts to manipulate migration timing might prove beneficial, such as by artificially cuing freshwater migration (e.g. by manipulating temperature or flow in rivers). In addition, it remains uncertain how endogenous clocks that are sensitive to photoperiod will respond to extreme and accelerated environmental change rather than to gradual changes (Kumar *et al*., 2010).

The rate of migration and timing of arrival are controlled by multiple factors, including intraspecific differences, particularly when early arrival confers fitness benefits. The rate of movement is likely to be related to optimization of energy use (e.g. Braithwaite *et al*., 2015), and stopover timing is probably controlled by circadian rhythms (Bartell and Gwinner, 2005; Sauman *et al*., 2005). However, birds that arrive in unfavourable conditions can depart and return later (Hahn *et al*., 2004). Glucocorticoids influence the arrival of migrating birds, with high corticosterone corresponding to earlier arrival (Lobato *et al*., 2010). The influence of glucocorticoids (e.g. corticosterone, cortisol) is important from a conservation perspective because they fluctuate throughout life to stimulate life-history transitions but can be manipulated by stress, which can potentially interfere with the expression of key life-history events and/or have measurable fitness consequences (Bush and Hayward, 2009). The exact location where individuals terminate migration depends on the species, with some exhibiting strict philopatry (e.g. Lea *et al*., 2015) and others simply seeking suitable habitat. Understanding the difference is non-trivial because philopatric species have less flexible migrations and can therefore be more susceptible to environmental change. Competition can also influence patterns of settlement that drive selection for the timing of migration (Møller, 1994; Drent *et al*., 2003). Arrival at breeding grounds coincides with reproductive maturation for most migratory species associated with territoriality, meaning that social groups or flocks of migrants (Ramenofsky and Wingfield, 2007) break, and individuals that were cooperative during migration become antagonistic.

Human interference can also affect the timing of animal migration, an important example of which is because of interactions with fisheries (Raby *et al*., 2015b). The mobility of many migratory species exposes them to fisheries and, indeed, many of the most important fisheries resources are migratory species, including salmonids, tunas, billfishes and cods. However, many non-teleost migrants are affected by fisheries as bycatch, particularly elasmobranchs, cetaceans, sea turtles and seabirds (Hall *et al*., 2000; Raby *et al*., 2011). For bycatch (which includes target species protected by harvest restrictions), interactions with fisheries are stressful and can have lethal and sublethal effects on fitness. Encounters with fisheries can cause physical damage to tissues (e.g. bleeding, barotrauma), reflex impairment from muscular exhaustion or insufficient oxygen delivery to the brain (Raby *et al*., 2015b), physiological disturbance in the muscle and blood (Cooke *et al*., 2013a) or external infection. After fisheries interactions, animals may require hours or days to restore homeostasis, during which time migration is delayed and predation risk is enhanced (Raby *et al*., 2014). In Atlantic salmon (*Salmo salar*) fisheries, release from recreational fisheries is associated with anomalous downriver movement, migratory delays and shorter migration (Lennox *et al*., 2015). Such delays or alterations to the migratory schedule can impair fitness of migrating animals, and research is ongoing in fisheries sectors to understand how migrations are affected by these human interactions and interferences. Efforts to reduce the impact of fisheries on aquatic resources rely on physiological knowledge and tools and include strategies for developing assessment protocols (e.g. Raby *et al*., 2012) and recovery strategies and tools (Farrell *et al*., 2001; Donaldson *et al*., 2013; Raby *et al*., 2015a; Robinson *et al*., 2015) for animals destined to be released by fishers. However, further efforts are needed to explore revival of non-teleost species that are often affected by fisheries.

## Synthesis

**Figure 2:**
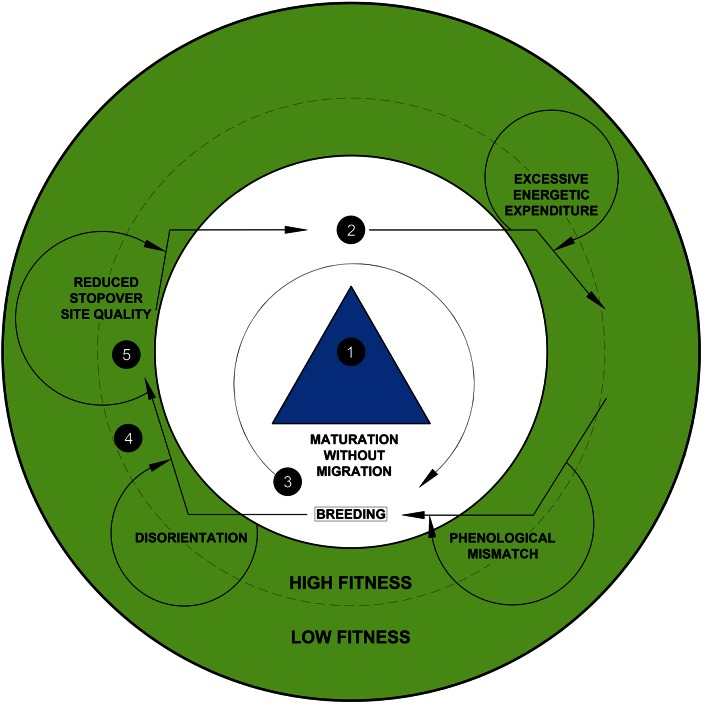
Migration is a physiologically challenging life-history stage, and there are many adaptations that animals have evolved for optimizing fitness (Fig. 1). Migratory species should optimally move through areas of high fitness in the migratory arena; however, anthropogenic change is altering the path through the migratory arena, which has consequences for lifetime fitness (represented by curved arrows). Some important conservation challenges are highlighted in this figure to demonstrate how they interface with fitness impairment. Conservation agendas must focus on mitigating such challenges to maintain high fitness of individual migrants and conserve migratory phenotypes, populations and species.

Although migration is ‘behaviour’, it is the manifestation of integrated physiological processes in animals (Berthold, 1996; Dingle, 2006). Migration incorporates considerable physiological adaptation as well as genetic, ontogenetic and morphological traits underlying a migratory syndrome (Dingle, 2006). Our overview of the physiological mechanisms controlling migration provides a mechanistic model of migration (see Fig. 1), explaining how and why it occurs, how it is regulated by animals, and some documented and potential changes to migration faced by animals in a changing world. Our physiological model of migration generalizes complex processes that sometimes have considerable variation among taxa but performs reasonably well in summarizing the important physiological variables that regulate migratory behaviour and its fitness end points. However, as a discipline focused on the cellular, biomechanical and biochemical processes of organisms, physiology has the capacity to provide more than information about individuals and can also generate knowledge that informs conservation (Tracy *et al*. 2006; Wikelski and Cooke, 2006; Cooke *et al*. 2013b; Madliger *et al*., 2016; Fig. 2).

Physiology is positioned to directly inform conservation efforts for managing migratory species (e.g. Cooke *et al*., 2012), including those that are obligatorily migrants as well as partial or facultative migrants (e.g. Chapman *et al*., 2012). Human activities and global change are altering the migratory arena and changing the balance of costs and benefits associated with migration such that there is the potential for various severe fitness impairments for migrating animals (Fig. 2). In some cases, ecosystem connectivity is threatened by development and sprawl, construction of dams, roads and tall buildings as well as the deterioration of the acoustic environment associated with anthropogenic noise (Tennessen *et al*., 2014). At temperate latitudes, springtime is advancing, deciduous plants are blooming earlier (Menzel, 2002), rain is replacing snow (Knowles *et al*., 2006), and resident species are advancing activity and reproduction (Gibbs and Breisch, 2001). In polar zones, sea surface temperatures are rising, ice cover is receding (Parmesan, 2006), primary production is changing (Smol *et al*., 2005), and atmospheric circulation patterns are altering winter conditions in the Northern hemisphere (e.g. polar vortexes in Eastern North America; Kim *et al*., 2015). These changes are challenges for animals and particularly for migratory species, which rely on many different habitats and geographical areas to complete their life history. Challenges that arise during migration can manifest as reduced fitness of migratory species (Fig. 2); therefore, the fundamental challenge posed to conservation is to understand and mitigate fitness impairments of migratory species in a changing world. Addressing conservation challenges will increasingly rely on understanding the physiological mechanisms that define migration (e.g. Cooke *et al*., 2012). There are increasing examples of physiology informing conservation initiatives (Lennox and Cooke, 2014), although the nascence of conservation physiology means that the success stories are limited, but growing (Madliger *et al*., 2016). Therefore, we foresee considerable potential for migratory physiology to be applied for conservation, and offer some relevant examples.

Some animals navigate using temporally unstable navigational cues, meaning that it is necessary to predict where the animals will be in order to monitor them and conserve critical habitat. Putman *et al.* (2013) suggested that the direction that sockeye salmon migrate through Queen Charlotte Straight (i.e. entering from the north or south direction) is predictable based on sea surface temperature and geomagnetic field drift. The ability to forecast the timing or direction of migration by understanding the physiological mechanisms used by fish to orient has conservation implications, particularly for fisheries and water resource management. Furthermore, anthropogenic noise can create perturbations in otherwise stable navigational cues, such as the electromagnetic field. Electromagnetic noise is emitted everywhere humans use electronic devices. Inability to orient during migration will decrease the likelihood of survival, increase the energetic cost of migration, delay arrival and, ultimately, impair fitness. Indeed, nocturnally migrating songbird populations are currently in dramatic decline, and the effect of anthropogenic electromagnetic noise on migratory physiology may be an underappreciated factor in their conservation. In a more general sense, the ability to predict migrations can be used to influence the management of humans (e.g. by managing vehicle operations, fisher behaviour, or dam operations or by reducing electromagnetic noise) or, alternatively, to influence the animals themselves (i.e. alter the route taken by migrants).

Using knowledge of the physiological basis for migratory path selection to prevent interactions with barriers is an active area of research for terrestrial and aquatic organisms. Improved understanding of sensory mechanisms (Blumstein and Berger-Tal, 2015) can be used in the development of management strategies, such as deflecting animals away from turbines or barriers using visual (lights), auditory (blasts) or somatosensory (bubble curtains) cues (Noatch and Suski, 2012). Switching to green coloured lights, which exclude short-wavelength red light that may affect cellular mechanisms associated with orientation, has reduced collisions between birds and ships and oilrigs by allowing birds to maintain migratory trajectory (Wiltschko *et al*., 1993; Poot *et al*., 2008). Moreover, ultraviolet lights might increase perceptibility of aircraft for the Canada goose (*Branta canadensis*) and reduce bird strike (Blackwell *et al*., 2012). In the future, there are further opportunities for lighting to be adapted for guiding migrants away from dangerous areas (e.g. turbines, blasting).

Disappearance of stopover habitat results in energetic depletion, competition for limited resources and disease transmission among migrants concentrated at a few high-quality sites. The exposure of an animal to an earlier, often spatially distinct site may influence reproductive success months later (e.g. Ceriani *et al*., 2015), thousands of kilometres away, even if the breeding grounds are in pristine condition (known as a carry-over effect; Norris and Taylor, 2006; O'Connor *et al.*, 2014). Unravelling how different stressors influence migratory animals is inherently challenging given the potential for these carry-over effects and the vast distances traversed by many animals (O'Connor and Cooke, 2015), making it necessary to conserve habitat along an entire migratory corridor. Strategically placed artificial stopover sites or areas where supplemental feed is deposited for migrants that are matched with energetically demanding portions of the migration could help to buffer the effects of habitat loss. Amano *et al.* (2007) simulated the use of supplemental feeding areas to reduce conflicts between migratory birds and agricultural crops but suggested that they would be unsuccessful unless carried out on a large scale. Supplemental feeding of birds and ungulates is common in some regions, and engineering feed to suit the nutritional requirements of migrants could provide a short-term solution for loss of stopover habitat, although in the long-term the preservation, remediation or replacement of stopover habitat is the only viable solution (Smith *et al*., 2015). Likewise, manipulating water levels in hydroelectric damn drawback areas may influence the extent to which migratory birds can refuel for their migration, although empirical studies of this have not been successful (Wagner *et al*., 2014).

Mistimed migrations have the potential to reduce the fitness of migratory animals by desynchronizing life-history events (including migration) and phenological processes that support those events, including favourable temperatures, vegetation bloom and insect emergence. It is difficult to use physiological knowledge or tools to counteract the likely effects of mismatched timing, particularly for animals that rely on fixed cues, such as photoperiod (Feder *et al*., 2010). The ability for animals to adapt their life history to climate change depends on evolutionary responses and phenotypic plasticity (Bradshaw and Holzapfel, 2008). However, one solution has been suggested as a response to climate warming, which is the use of artificial freshets for manipulating the timing of river entry for anadromous fishes (Huntsman, 1942). Once in the river, fish may find microhabitat to buffer high water temperatures; by entering earlier in the spring, they can avoid active migration during high summer temperatures when cardiorespiratory systems become strained to deliver sufficient oxygen (Eliason *et al*., 2013). Migrating fish may be less prone to significant mortality en route if their migrations are completed early in the season when water temperatures are lower. When migration is sufficiently disturbed that animals can no longer move, facilitated migration has shown promise for maintaining a population of migratory lobster (Green *et al*., 2010). Although manual transport is not a viable option for most species, it may be possible to facilitate migration of some species, particularly around physical barriers (e.g. around dams using specialized transport trucks; Sigourney *et al*., 2015; hydraulic pumps, such as the ‘salmon cannon’; Mesa *et al*., 2013). Moreover, Hartup *et al*. (2004) suggested that captive-rearing of greater sandhill cranes (*Grus canadensis*) and then training them to migrate from Wisconsin to Florida using ultralight aircraft was viable on the basis of their normal faecal corticosterone profiles during the assisted migration. Selective breeding of individuals that are adequately adapted to specific changing environmental conditions is a drastic measure that could improve stocks of migratory species via enhancement programmes. For example, there is evidence that different populations or stock complexes of salmon are better equipped for climate change as a result of aerobic acclimation to high water temperatures (Eliason *et al*., 2011). Stock enhancement programmes have the unique platform to proliferate these physiological phenotypes if extant populations do not sufficiently track climate change naturally. Of course, the broader ecological consequences of such intervention would have to be critically addressed before the implementation of such measures could be considered (e.g. Ford *et al*., 2015).

## Conclusions

In an era of substantial human-induced rapid environmental change, science is increasingly focused on generating solutions to conservation problems (Soulé, 1985). There is imminent concern that climate change will affect migratory species (Robinson *et al*., 2009), but it is important to recognize that migration is a behaviour that has evolved to cope with extreme environmental variability and has persisted and continued to evolve over millions of years of global change. Indeed, it should be anticipated that an era of continued change would be met with further evolution and adaptation by these migratory species (Visser 2008). As a result, it is somewhat tempting to predict that many mobile species will be able to compensate for changes in environmental conditions by adjusting their migratory strategies via plasticity or microevolution. Already, there are examples of animals adapting their migratory phenotypes to account for climatic changes (Berthold *et al*., 1992; Able and Belthoff, 1998; Juanes *et al*., 2004), and models predict further adaptations, including smaller size (Clark *et al*., 2012) and advanced maturation (i.e. without migration; Morita *et al*., 2014), to cope with changing fitness landscapes (Fig. 2). In addition, improved conditions for feeding during prolonged temperate summers have the potential to decrease the need for migration of some species (Brodersen *et al*., 2008).

In the midst of unprecedented change, a comprehensive understanding of how oncoming disturbances will affect ecosystems remains elusive and requires better baseline information about animal physiology. Migratory species are an important point of conservation emphasis given their ecological and economic importance. Madliger *et al*. (2016) demonstrated that conservation physiology is transitioning from a theoretical discipline to one that is materializing in conservation action, and although success stories for migratory species in the published literature are not yet common (but see Cooke *et al*., 2012), we anticipate a growing role for this synergy in the conservation of migratory species. However, our review has demonstrated that there are still key knowledge gaps related to conservation physiology of migrating animals and that there is a disproportionate focus on migratory birds and teleost fishes in the conservation physiology literature. These challenges are likely to be due in large part to difficulties in studying highly mobile animals across scales. Tracking, sampling or holding small-bodied insects or large-bodied and cryptic whales to gain insight on mechanisms of enormous, population-scale movements are challenges that must be overcome through the development and implementation of new techniques for gaining physiological insight (see Jachowski and Singh, 2015). Indeed, the complexity of biological systems, the inherent dynamic nature of the environment and the scale at which many migrations occur and associated multiple threats operate complicate links between physiological stressors, stress responses and fitness consequences (Bush and Hayward, 2009). Nonetheless, we submit that further integration of basic and applied physiological research, tools, knowledge and concepts (Blumstein and Berger-Tal 2015; Jachowski and Singh, 2015; Sopinka *et al*., 2015) with behavioural ecology and conservation science (see Cooke *et al*., 2014) will be important and necessary for developing and refining strategies to meet conservation and management objectives related to migratory species in a changing world.

## Funding

R.J.L. and J.M.C. were supported by the Natural Science and Engineering Research Council of Canada (NSERC) Graduate Fellowships. S.J.C. was supported by NSERC (via a Discovery Grant, Strategic Grants and both NSERC HydroNet and Ocean Tracking Network Canada) and the Canada Research Chairs programme.
